# ﻿First mitogenomic characterization of *Macromotettixoides* (Orthoptera, Tetrigidae), with the descriptions of two new species

**DOI:** 10.3897/zookeys.1195.112623

**Published:** 2024-03-14

**Authors:** Jieling Luo, Rongjiao Zhang, Weian Deng

**Affiliations:** 1 Key Laboratory of Ecology of Rare and Endangered Species and Environmental Protection, Guangxi Normal University, Ministry of Education, Guilin, Guangxi 541006, China Guangxi Normal University Guilin China; 2 School of Chemistry and Bioengineering, Hechi University, 546300, Yizhou, Guangxi China Hechi University Yizhou China; 3 Guangxi Key Laboratory of Rare and Endangered Animal Ecology, Guangxi Normal University, Guilin, Guangxi 541006, China Guangxi Normal University Guilin China; 4 College of Life Science, Guangxi Normal University, Guilin, Guangxi 541004, China Hechi University Yizhou China

**Keywords:** China, *Formosatettix*, Metrodorinae, mitochondrial genome, phylogeny, taxonomy, Tetriginae

## Abstract

Classification of species is commonly based on morphological, molecular, and distribution depending on the taxa. *Macromotettixoides* Zheng, Wei & Jiang, 2005 and *Formosatettix* Tinkham, 1937 are both wingless types of Tetrigidae with extremely similar morphological characteristics, and in the current taxonomic system they are placed in two different subfamilies, Metrodorinae and Tetriginae, respectively. It is difficult to clearly identify the species of these two genera by morphological characteristics, and molecular data is often needed to assist identification. Here, the complete mitogenomes of two new species were sequenced and assembled, with that of *Macromotettixoidesorthomargina*. Molecular data of species of *Formosatettix* were used to test the monophyly of *Macromotettixoides* and to re-assess the generic characters, and also to test whether *Macromotettixoides* belongs to the Asian Metrodorinae or Tetriginae. Furthermore, mitochondrial characteristics were analyzed and the phylogeny of the Tetrigidae reconstructed based on mitochondrial protein-coding genes (PCGs). The results indicated that the two new species were clustered with *Macromotettixoides* rather than *Formosatettix*, and the anterior margin of the fastigium and pronotum of the two new species usually had the humeral angle different from that of *Formosatettix*. Therefore, after integrating morphological and molecular data, the two new species were placed in the genus *Macromotettixoides*, *M.maoershanensis***sp. nov.** and *M.brachycorna***sp. nov.** Finally, a phylogenetic reconstruction supported *Macromotettixoides* being assigned to Tetriginae rather than Metrodorinae, in contrast to the previous classification of this genus.

## ﻿Introduction

Tetrigidae is a family of Orthopteran insects in the superfamily Tetrigoidea. The pygmy grasshoppers are an ancient group of Orthoptera with a relatively uniform body structure (Holst 1986; Zhao et al. 2016). However, the polymorphism within species, including different wing morphs and color variation, poses several challenges to classification. In order to effectively identify species, it is therefore necessary to combine as many different types of data as possible.

Mitogenomes are stable in structure and composition; they have maternal inheritance and less recombination (Simon et al. 2006; Cameron 2014). Mitochondrial genomes have been widely used to study species lineages, biological evolution, and species classification. The mitochondrial genomes of insects are generally between 15k–20k bp in size, containing 22 tRNAs, 13 protein-coding genes (the ND series, the CO series, the ATP series, and *Cytb*, two rRNAs, and a control region (or A+T rich region)) (Clary and Wolstenholme 1985; Cheng et al. 2000). The transposition of *trnD* and *trnK* is common in Caelifera (Boore 1999).

To date, among the Tetrigidae, only 35 species have their mitochondrial genomes published in GenBank. Additionally, many Tetrigidae have known COI sequences, but these are not adequate for deep phylogeny, only for species identification (Fang et al. 2010; Kasalo et al. 2023). More mitochondrial genomes should be sequenced in the future to help researchers further investigate the evolutionary history of the Tetrigidae.

*Macromotettixoides* Zheng, Wei & Jiang, 2005 (Zheng et al. 2005) is a genus of the subfamily Metrodorinae, for which a total of 24 species have been recorded as endemic to China. *Macromotettixoides* is very similar to *Formosatettix* Tinkham, 1937 (subfamily Tetriginae). The differences between the two genera occur in the vertex and the pronotum. In *Formosatettix*, the fastigium of the vertex in dorsal view surpasses the anterior margin of the eyes; the anterior margin of the fastigium is generally arched or angularly projecting, sometimes straight; the pronotum is usually without a humeral angle, with the posterior angles of the lateral lobes turned downwards, and the apex of the posterior angles obtuse and rounded. In *Macromotettixoides*, the fastigium of the vertex in dorsal view does not surpass the anterior margin of the eyes; the pronotum is with humeral angles; the posterior angles of the lateral lobes are usually turned outwards, and the apex of the posterior angles is truncated or sometimes slightly obtuse and rounded. In reality, the classification and identification of the two genera is relatively difficult, and sometimes molecular methods are needed for determination.

In this study, we collected two new species in Guangxi from China and discovered that they shared traits with both genera *Macromotettixoides* and *Formosatettix*. The two new species are similar to *Formosatettix* in that the anterior margin of the fastigium is angularly projecting and the posterior angles of pronotum turned downwards, and the apex of the posterior angles obtuse and rounded, while the pronotum is with humeral angles are similar to *Macromotettixoides*. Therefore, based solely on their morphology, it was difficult to classify them to any genus. The purposes of this study were to sequence the complete mitochondrial genomes of two new species and *Macromotettixoidesorthomargina* Wei & Deng, 2023, to examine their phylogenetic positions and relationships within the genus *Macromotettixoides* and *Formosatettix*, and to describe and illustrate the two new species from China.

## ﻿Materials and methods

### ﻿Taxon sampling

Specimens of *M.orthomargina*, *M.maoershanensis* sp. nov., and *M.brachycorna* sp. nov. were selected as representatives of the genus *Macromotettixoides*. (1) *M.orthomargina*, *n* = 3, collected at Lingshan, Mianning County, Sichuan Province, China; 23 June 2020; (2) *M.brachycorna* sp. nov., *n* = 3, collected at Jiuwanshan National Nature Reserve, Huanjiang Country, Guangxi, China; 25°11'41"N, 108°38'51'E; 29 July 2022; (3) *M.maoershanensis* sp. nov., *n* = 14, collected at Maoershan National Nature Reserve, Xing’an County, Guangxi, China; 25°51′35″N, 110°29′34″E; 12 July 2021. The specimens were preserved in 100% anhydrous ethanol (Xilong Scientific, Sichuan, MA, China) and stored in the refrigerator at -20 °C in the Key Laboratory of Ecology of Rare and Endangered Species and Environmental Protection, Ministry of Education of Guangxi Normal University (**MEGNU**). All photographs were taken using the Keyence VHX-5000 (Keyence Corporation, Osaka, Japan) and edited in Adobe Photoshop 23.0.0.

To clarify the taxonomic status of *M.maoershanensis* sp. nov. and *M.brachycorna* sp. nov., we combined the mitochondrial genome data assembled in the laboratory and the complete mitochondrial genome data of Tetrigidae from GenBank, representing one family, five subfamilies, 23 genera, and 36 species in total (Table 1). *Mirhipipteryxandensis* Günther, 1969 in Tridactyloidea (NC_028065) was selected as the outgroup. A phylogenetic tree of the Tetrigidae was constructed based on Bayesian inference (BI) and maximum likelihood (ML) methods.

**Table 1.** Accession numbers and references of the mitogenomes of Tetrigidae included in this study.

Subfamily	Species	Accession number	Reference
Tripetalocerinae	*Tripetaloceroidestonkinensis*	MW770353	Zhang et al. 2021
Batrachideinae	*Saussurellaborneensis*	MZ169555	Deng et al. 2021
Metrodorinae	*Bolivaritettixlativertex*	MN083173	Chang et al. 2020
	*Bolivaritettixsikkinensis*	MN083174	Yang 2017
	*Bolivaritettixyuanbaoshanensis*	KY123121	Yang 2017
	*Mazarrediaconvexa*	MN938924	Li et al. 2020c
Criotettigini	*Criotettixjaponicus*	MT162542	Li et al. 2021a
Scelimeninae	*Falconiuslongicornis*	MT162543	Li et al. 2021a
	*Paragavialidiumhainanense*	NC_071831	
	*Paragavialidiumsichuanese*	MT162549	Li et al. 2021a
	*Scelimenamelli*	MW722938	Li et al. 2021b
	*Zhengitettixcurvispinus*	MT162544	Li et al. 2021a
Thoradontini	*Eucriotettixoculatus*	MN083176	Chang et al. 2020
	*Loxilobusprominenoculus*	MT162545	Li et al. 2021a
	*Thoradontanodulosa*	MT162547	Li et al. 2021a
	*Thoradontaobtusilobata*	KY798414	Lin et al. 2017
	*Thoradontayunnana*	OP805341	
Tetriginae	*Alulatettixyunnanensis*	NC_018542	Xiao et al. 2012a
	*Coptotettixlongjiangensis*	KY798413	Lin et al. 2017
	*Coptotettixlongtanensis*	OK540319	
	*Ergatettixserrifemora*	MN938923	Chang et al. 2020
	*Ergatettixdorsifera*	NC_046540	Chang et al. 2020
	*Euparatettixbimaculatus*	NC_046541	Chang et al. 2020
	*Euparatettixvariabilis*	NC_046542	Chang et al. 2020
	*Formosatettixqinlingensis*	KY798412	Lin et al. 2017
	*Macromotettixoidesbrachycorna* sp. nov.	OR003899	This study
	*Macromotettixoidesmaoershanensis* sp. nov.	OR030790	This study
	*Macromotettixoidesorthomargina*	OR030789	This study
	*Systolederusanhuiensis*	OP113951	
	*Systolederusbashanensis*	NC_063118	Li et al. 2021
	*Systolederushainanensis*	NC_063117	Li et al. 2021
	*Systolederusnigropennis*	MN938922	Li et al. 2020b
	*Systolederusspicupennis*	MH791445	
	*Tetrixjaponica*	NC_018543	Xiao et al. 2012b
	*Tetrixruyuanensis*	NC_046412	Chang et al. 2020
Outgroup	*Mirhipipteryxandensis*	NC_028065	Song et al. 2015

### ﻿Sequencing, assembly, and annotation

All muscle tissues of each sample were extracted using a TlANamp Genomic DNA Kit (Tiangen Biotech, Beijing, China), and the extracted samples were sent to Berry Genomics (Beijing, China) for genomic sequencing using Next Generation Sequencing (NGS). The remaining specimens were deposited as voucher specimens at the Guangxi Normal University. Separate 350-bp insert libraries were created from the whole genome DNA and sequenced using the Illumina HiSeq X Ten sequencing platform. A total of 5 Gb of 150-bp paired-end (PE) reads were generated in total for each sample. The mitogenome sequences were assembled using NOVOPlasty 4.2.1 and annotated using the MITOS Web Server (http://mitos2.bioinf.uni-leipzig.de/index.py, accessed on 17 March 2023; Donath et al. 2019). The annotated mitogenome sequences were checked in CLC Genomics Workbench 12.0.0, MEGA 11.0.1, and Geneious Prime 11.0.15. The maps of the mitogenomes were generated using the Proksee website (https://proksee.ca, accessed on 19 December 2023, Grant et al. 2023). The secondary structures of the RNA encoding genes predicted in MITOS were visualized manually using Adobe Photoshop 23.0.0. All sequences generated from this study were deposited in GenBank (for accession numbers see Table 1).

The base compositions, G–C- and A–T-skews, and codon usages were calculated in PhyloSuite v. 1.2.3. The formulas used to calculate the skews of the composition were (A–T) / (A+T) for the A–T-skew and (G–C) / (G+C) for the G–C-skew.

### ﻿Phylogenetic analyses

To systematically understand the phylogenetic relationships of *M.orthomargina* and the two new species, the mitochondrial genomes of the three species obtained in the laboratory and the mitogenomes of 36 species taken from GenBank were used to construct a phylogeny of the Tetrigidae, and *Mirhipipteryxandensis* of the Tridactyloidea was selected as the outgroup. The analysis was performed using PhyloSuite 1.2.3. Redundant sequences were removed, and protein-coding genes in the mitochondrial genome were extracted and aligned in batches with MAFFT (Katoh and Standley 2013). The aligned sequences were concatenated. ModelFinder (Kalyaanamoorthy et al. 2017) was used to select the best-fit model using AICc and BICc standards. The best-fitting model was used for the phylogenetic analyses of the mitochondrial PCGs (Table 2).

**Table 2.** Best-fitting models used for phylogenetic analyses of the mitochondrial PCGs dataset.

Information Criterion for model selection	Best model	Partition names
AICc	GTR+F+R4	cox3_mafft, nad4L_mafft, nad4_mafft
	TIM+F+I+I+R5	nad1_mafft
	GTR+F+I+I+R5	cox1_mafft, nad5_mafft
	TIM2+F+I+I+R4	cox2_mafft
	TIM3+F+R5	atp6_mafft, nad2_mafft
	TIM2+F+R4	cytb_mafft, nad3_mafft
	TIM3+F+I+I+R4	nad6_mafft
	TN+F+I+I+R3	atp8_mafft
BIC	GTR+F+I+G4	atp6_mafft, cox2_mafft, cytb_mafft, cox1_mafft, cox3_mafft, nad1_mafft, nad4L_mafft, nad4_mafft, nad2_mafft, nad3_mafft, nad5_mafft, nad6_mafft, atp8_mafft

Bayesian inference phylogenies were obtained using MrBayes v. 3.2.7a (Ronquist et al. 2012) under the GTR+F+I+G4 model. The analysis was run for 4000006 generations, two parallel runs, sampling every 100 generations, and the first 25% generations were discarded as burn in, whereas the remaining samples were used to summarize Bayesian posterior probabilities (PP). Support for each branch was derived from the posterior probabilities (PP) observed on the majority-rule consensus.

The maximum likelihood phylogenies were inferred using IQ-TREE v. 2.2.0 (Nguyen et al. 2015) under the Edge-linked partition model with 5000 ultrafast bootstrap replicates (Minh et al. 2013). The resulting phylogenetic tree was further edited on the iTOL website (https://itol.embl.de/itol.cgi, accessed on 20 March 2023; Letunic and Bork 2021).

## ﻿Results

### ﻿Characteristics of newly sequenced mitogenomes

**Figure 1.**
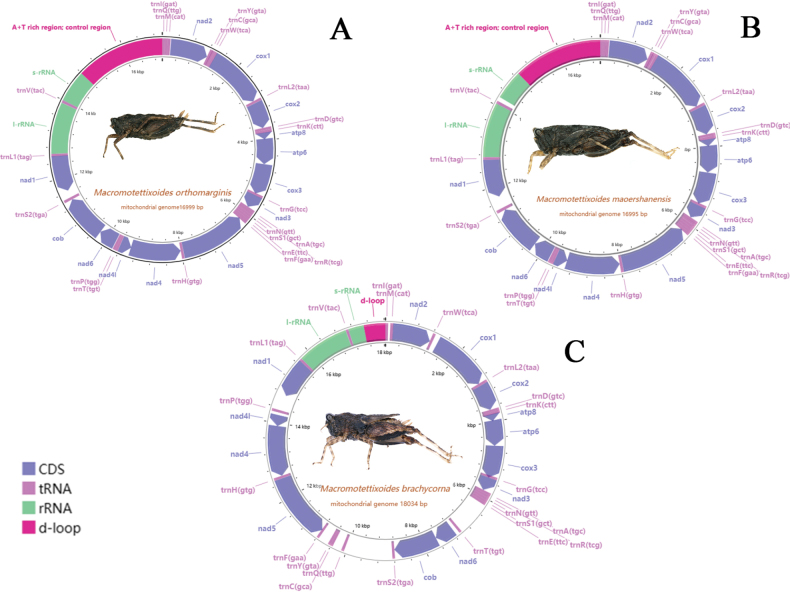
Circular map of the mitogenomes **A***M.orthomargina***B***M.maoershanensis* sp. nov. **C***M.brachycorna* sp. nov.

In this study, the mitochondrial genomes of *M.orthomargina*, *M.brachycorna* sp. nov., and *M.maoershanensis* sp. nov. were all circular molecules, with total lengths of 16,995 bp, 18,034 bp, and 16,999 bp, respectively (Fig. 1). The structures of the three newly sequenced *Macromotettixoides* species mitogenomes were the same as those of the mitochondrial genomes of other metazoan animals (Bernt et al. 2013), with 13 protein-coding genes, 22 tRNAs, 2 rRNAs and control regions rich in A+T bases. Among the 13 protein-coding genes, *nad1*, *nad4*, *nad4L*, and *nad5* were located on the N strand, while the other genes (*nad2*, *nad3*, *nad6*, *cox1*, *cox2*, *cytb*, *atp6*, and *atp8*) were located on the J strand. In all mitochondrial genes, 14 genes were located on the minority strand, and 23 genes were located on the majority strand.

**Figure 2.**
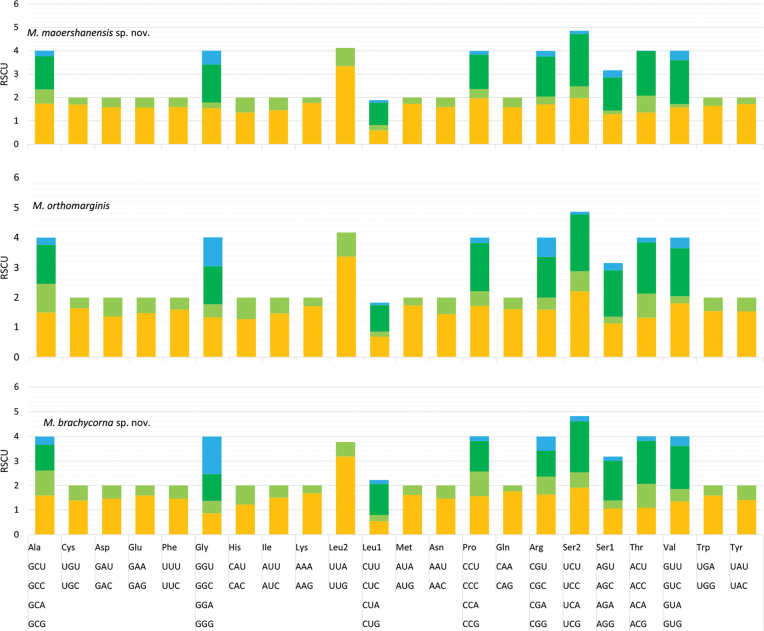
Relative synonymous codon usage (RSCU) of the mitochondrial genomes of three species in *Macromotettixoides*.

The gene arrangements of the newly sequenced mitochondrial genomes were similar to those of other species of Tetrigidae (Lin 2014). The base composition was A+T-biased, and the proportions of A+T content were 73.1% (*M.orthomargina*), 71.4% (*M.brachycorna* sp. nov.), and 73.7% (*M.maoershanensis* sp. nov.). The AT-skews were 0.1997 (*M.orthomargina*), 0.158 (*M.brachycorna* sp. nov.), and 0.1967 (*M.maoershanensis* sp. nov.), and the GC-skews were –0.2937 (*M.orthomargina*), –0.274 (*M.brachycorna* sp. nov.) and –0.2966 (*M.maoershanensis* sp. nov.) (Table 3). The relative synonymous codon usage (RSCU) values of the mitogenome were summarized (Fig. 2). The codon distribution analysis showed that the two codons UUA (Leu2) and UCA (Ser2) were the most frequently used in *M.maoershanensis* sp. nov. and *M.brachycorna* sp. nov. The codons of UUA (Leu2) and UCU (Ser2) in *M.orthomargina* were the most frequently used. The frequency of the codons ending with A/U was much higher than with G/C, suggesting that the AU composition at the third position of codons had a positive influence on the nucleotide AT (or AU) bias of the PCGs in *Macromotettixoides*.

**Table 3.** Nucleotide composition of the mitogenomes of *Macromotettixoides*.

Regions	A%	G%	AT%	AT-skew	GC-skew
Full genome	41.4/44.1/43.9	10.4/9.2/9.5	71.4/73.7/73.2	0.158/ 0.198/0.200	–0.274/–0.296/–0.293
PCGs	31.0/ 31.9/31.2	14.1/13.2/13.6	69.4/72.3/71.2	–0.108/–0.117/–0.124	–0.079/–0.046/–0.051
1^st^ codon position	34.0/34.8/34.3	18.5/17.9/18.1	67.1/69.0/68.9	0.013/0.008/–0.004	0.128/0.152/0.163
2^nd^ codon position	20.3/20.3/20.4	14.8/14.7/14.7	65.6/ 65.9/66.2	–0.382/–0.384/–0.384	–0.140/–0.135/–0.130
3^rd^ codon position	38.6/40.5/38.9	9.0/7.1/8.1	75.4/81.7/78.6	0.023/–0.009/–0.01	–0.270/–0.217/–0.240
rRNAs	40.8/41.8/41.6	12.7/12.2/12.5	74.2/75.0/74.0	0.100/ 0.115/0.124	–0.017/–0.026/–0.038
tRNAs	28.8/27.3/27.6	16.1/15.9/16.4	75.2/76.9/76.1	–0.234/–0.290/–0.274	0.296/0.374/0.372
CR	36.2/46.3/48.1	13.1/9.6/8.0	69.4/79.2/80.0	0.040/0.170/0.203	–0.140/–0.077/–0.200

Note: Data are given as *M.brachycorna* sp. nov./ *M.maoershanensis* sp. nov./ *M.orthomargina*. CR, control region.

As in most pygmy grasshoppers, ATN was the initiation codon of *M.orthomargina*, *M.brachycorna* sp. nov., and *M.maoershanensis* sp. nov., with ATG being the most frequently used (Table 4). The initiation codons of *ND2*, *ND3*, and *ND6* in *M.brachycorna* sp. nov. were different from those of the other two species. *ND2*, *ND3*, and *ND6* in *M.brachycorna* sp. nov. initiated from ATA, ATC, and ATG respectively, whereas *ND2*, *ND3*, and *ND6* in *M.maoershanensis* sp. nov. and *M.orthomargina* initiated from ATT, ATA, and ATA, respectively. The termination codon was the typical TAN, in which TAA was used most frequently, followed by TAG. The termination codon of *ND4* was TAA (*M.brachycorna* sp. nov.) and TAG (*M.maoershanensis* sp. nov. and *M.orthomargina*).

**Table 4.** Initiation and termination codons of PCGs of the newly sequenced complete mitogenomes.

PCGs	Initiation codons			Termination codons		
	*M.maoershanensis*	*M.brachycorna*	*M.orthomargina*	*M.maoershanensis*	*M.brachycorna*	*M.orthomargina*
ATP6	ATG	ATG	ATG	TAA	TAA	TAA
ATP8	ATG	ATG	ATG	TAA	TAA	TAA
COX1	ATC	ATC	ATC	TAA	TAA	TAA
COX2	ATG	ATG	ATG	TAA	TAA	TAA
COX3	ATG	ATG	ATG	T	T	T
CYTB	ATG	ATG	ATG	TAA	TAA	TAA
NAD1	ATT	ATT	ATA	TAA	TAA	TAA
NAD2	ATT	ATA	ATT	TAA	TAA	TAA
NAD3	ATA	ATC	ATA	TAG	TAG	TAG
NAD4	ATG	ATG	ATG	TAG	TAA	TAG
NAD4L	ATT	ATT	ATT	TAA	TAA	TAA
NAD5	ATG	ATG	ATG	T	TA	T
NAD6	ATA	ATG	ATA	TAA	TAA	TAA

Comparing the AT content of the mitochondrial genomes’ PCGs, rRNAs, tRNAs, and the control regions of Tetrigidae (Suppl. material 1: table S1), there was not significantly difference in the lengths of PCGs and 16S rRNA within the same genus, such as *Macromotettixoides*, *Systolederus* Bolívar, 1887, and *Thoradonta* Hancock, 1909. The lengths of tRNAs and 12S rRNA varied in the different genera. The difference in the total length of the mitochondrial genes among different species is mainly due to the difference in the control region length, and there were also differences in the AT content among different genes within the same genus. The PCGs of *Bolivaritettix* is shorter compared to other species, due to its lack of *nad4*. The overall AT content of *Scelimena* and *Tripetaloceroides* was relatively low, while the AT content of *Systolederus* was relatively high.

Comparison of the codons of PCGs in Tetrigids (Suppl. material 1: tables S2, S3) showed that most species mainly started with ATN, with a small number using TTG, GTG, ACA, AAA, etc. as the starting codons. Among them, the *cox1* of many species started with ACA and AAA. The starting codons of the same genus were not exactly the same, such as these genera *Macromotettixoides* and *Paragavialidium* Zheng, 1994, *Systolederus*, and *Thoradonta*. The termination codons of PCGs were typical TAG or TAA, with *cox1*, *cox3*, and *nad*5 mainly terminate with incomplete codons, T or TA. There were certain differences in the termination codons among species of the same genus. Some studies proposed that the incomplete T-termination codons can form complete termination codons through polyadenylation during mRNA processing (Weng et al. 2022).

**Figure 3.**
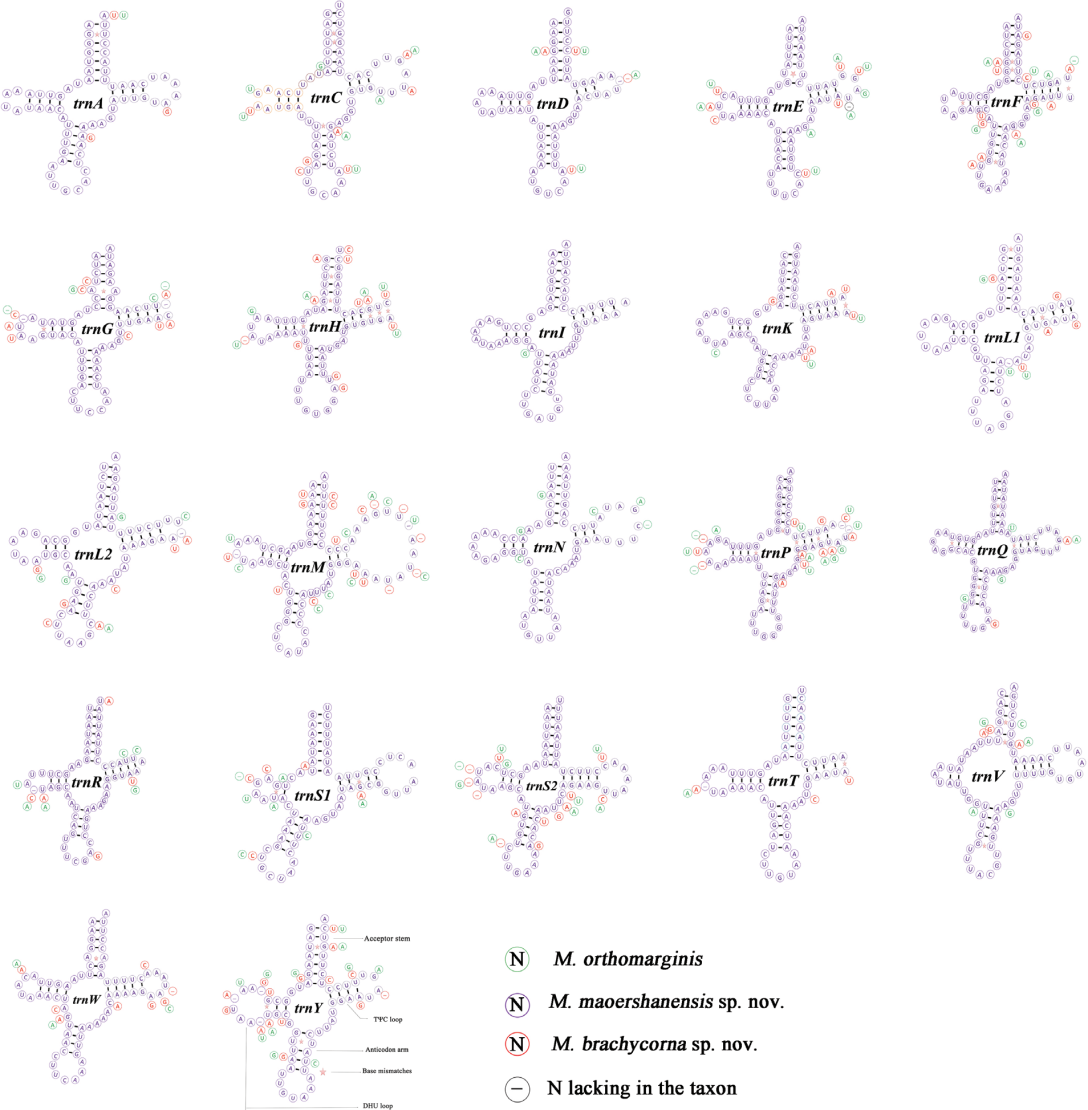
Secondary structure for the tRNAs of three species in *Macromotettixoides*.

There was little difference in the lengths of all tRNAs in the newly sequenced mitochondrial genomes of *M.orthomargina* and *M.maoershanensis* sp. nov. All secondary structures of the tRNAs of the three species could be folded into a typical clover structure (Fig. 3), except for trnS1 of *M.orthomargina* and *M.brachycorna* sp. nov. The *trnS1* of *M.orthomargina* and *M.brachycorna* sp. nov. lacked the DHU arm. The types and number of tRNA mismatches differed between *M.orthomargina* and *M.maoershanensis* sp. nov. The mismatch of A–A occurred in *trnW* and *trnG*; A–G occurred in *trnG* and *trnF*; A–C occurred in *trnG* and *trnS2*; and C–U only occurred in *trnM* (Table 5). The mismatch of U–U existed in *trnE*, *trnF*, *trnR*, and *trnY*. The G–U mismatch occurred most frequently, but it did not appear in *trnI*, *trnW*, *trnR*, *trnE*, or *trnT*, and there were six G–U mismatches (Table 6).

**Table 5.** Total numbers of different types of base mismatches in tRNAs of the three newly sequenced mitogenomes.

Species	A–A	A–G	A–C	G–U	C–U	U–U
*M.orthomargina*	1 (*trnW*)	1 (*trnG*)	1 (*trnS2*)	34		2 (*trnY*, *trnE*)
*M.brachycorna* sp. nov.	2 (*trnW*, *trnG*)	1 (*trnF*)	2 (*trnG*)	35	1 (*trnM*)	4 (*trnY*, *trnR*, *trnE*, *trnF*)
*M.maoershanensis* sp. nov.	2 (*trnW*, *trnG*)			35		2 (*trnY*, *trnE*)

**Table 6.** Distribution of G–U base mismatches in tRNAs of *M.orthomargina*, *M.brachycorna* sp. nov., and *M.maoershanensis* sp. nov.

Transfer RNA	*M.orthomargina*	*M.brachycorna*	*M.maoershanensis*	Transfer RNA	*M.orthomargina*	*M.brachycorna*	*M.maoershanensis*
*trnI*	0	0	0	*trnR*	0	0	0
*trnQ*	5	4	4	*trnN*	1	1	0
*trnM*	0	1	0	*trnS1*	0	0	2
*trnW*	0	0	0	*trnE*	0	0	0
*trnC*	3	3	3	*trnF*	5	3	5
*trnY*	3	3	2	*trnH*	3	3	5
*trnL2*	1	1	0	*trnT*	0	0	0
*trnD*	1	1	1	*trnP*	5	4	6
*trnK*	0	1	0	*trnS2*	0	1	0
*trnG*	1	1	1	*trnL1*	3	3	2
*trnA*	1	2	1	*trnV*	2	3	3

### ﻿Phylogeny

**Figure 4.**
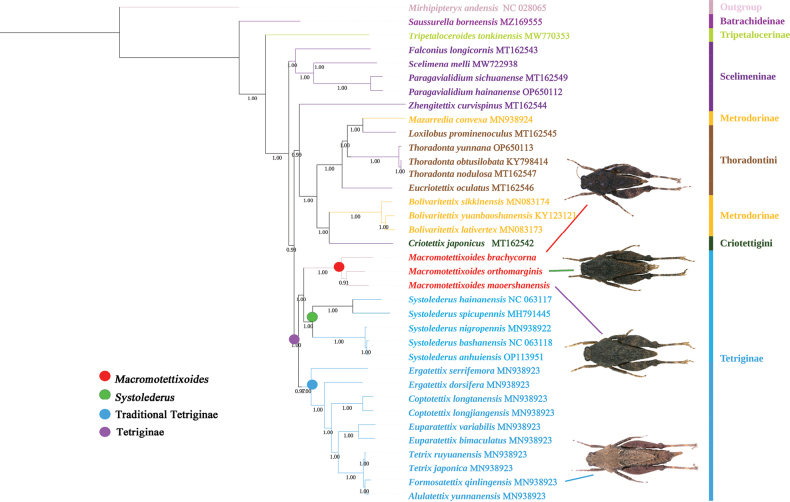
BI tree resulting from the analysis of 13 PCGs of mitochondrial genomes in the Tetrigidae.

**Figure 5.**
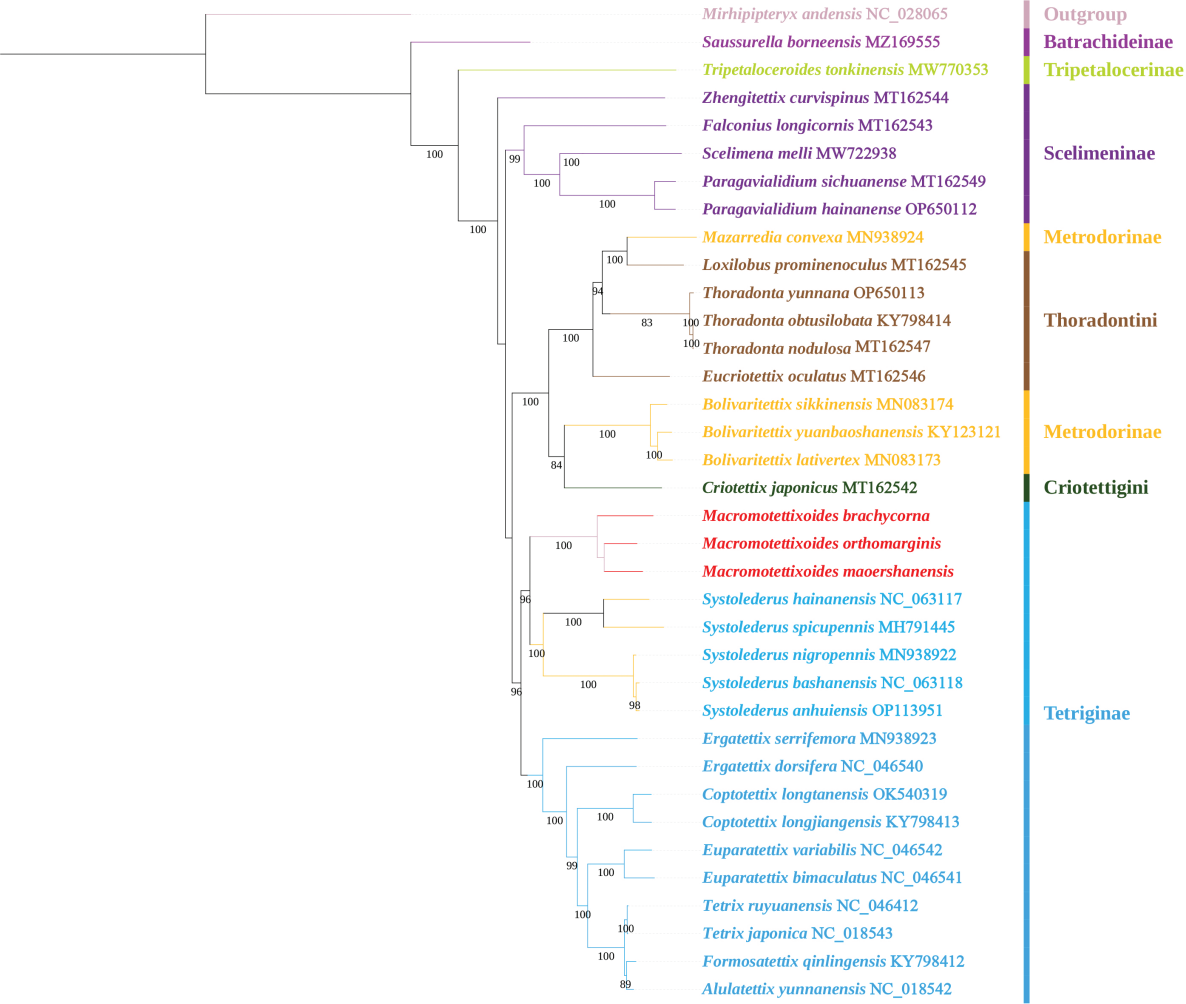
ML tree resulting from the analysis of 13 PCGs of mitochondrial genomes in the Tetrigidae.

This study supported the non-monophyly of Metrodorinae and Scelimeninae (Figs 4, 5), and this was highly supported by the BI analysis (PP > 0.90). However, there was only one species’ datum for Tripetalocerinae and Batrachideinae, and thus their monophyly could not be determined. The monophyly of most species in the same genus as *Bolivaritettix* Günther, 1939, *Paragavialidium*, *Thoradonta*, *Coptotettix* Bolívar, 1887, *Euparatettix* Hancock, 1904, and *Tetrix* Latreille, 1802 was supported in the BI tree, the same as in Wang et al. (2021).

In this study (Figs 4, 5), the phylogenetic reconstruction suggested that the two new species were clustered into a monophyletic group with *M.orthomargina* rather than with *F.qinlingensis* (PP = 1.00). Although the two new species were very similar to *Formosatettix* in the posterior angles of the lateral lobes being turned downwards and the apex of the posterior angles being obtuse and rounded, they were not classified in the genus *Formosatettix* but rather in *Macromotettixoides*. Therefore, we named the two new species as *M.maoershanensis* sp. nov. and *M.brachycorna* sp. nov. In the phylogenetic analysis, *Macromotettixoides* was found to be a sister group to *Systolederus*, and the clades of *Macromotettixoides* and *Systolederus* were clustered with the traditional Tetriginae. This agreed with the findings of Devriese and Husemann (2023) who proposed that the species of *Teredorus* in Indo-Malaysia be placed in *Systolederus*, and *Systolederus* should be placed in Tetriginae based on morphology and distribution. We also support the temporary classification of *Systolederus* within the subfamily Tetriginae, but this subfamily is polyphyletic and in need of revision. Therefore, the phylogenetic trees indicated that *Macromotettixoides* (lateral lobes of pronotum produced forward; end of posterior angles truncated) is a genus of Tetriginae rather than Metrodorinae.

Previous studies on the classification of Metrodorinae or Tetriginae were based on the morphology of the posterior angles of lateral lobes of pronotum (turned downwards, rounded, or produced forward, truncated), but some investigations suggested that distinguishing between Metrodorinae and Tetriginae based on the shape of the lateral lobes of the pronotum was unreliable (Adžić et al. 2020; Devriese and Husemann 2023). Therefore, it is necessary to use several morphological features, alongside molecular evidence, to correctly distinguish the subfamilies in Tetrigidae.

### ﻿Taxonomy

#### ﻿ Macromotettixoides maoershanensis sp. nov.

Taxon classificationAnimaliaOrthopteraTetrigidae

AB2D963A-1B2F-55DB-8ED1-6B5F6B54C934

https://zoobank.org/EB83298E-FE9B-4536-9D58-60177DF22802

Figs 6 , 7

##### Materials examined.

***Holotype***: China ♀; Guangxi, Guilin, Xing’an Country, Gaozhai; 25°51'35"N, 110°29'34"E; alt. 652.7 m; 12.VII.2021; Wei’an Deng, Chaomei Huang leg. ***Paratypes***: China 5♀, 1♂; Guangxi, Guilin, Xing’an Country, Gaozhai; 25°51'35"N, 110°29'34"E; alt. 652.7 m; 20.VII.2022, Jieling Luo, Chaomei Huang leg. 6♀, 2♂; Guangxi prov., Guilin, Longsheng Country, Hongtan; 25°36'34"N, 109°57'55"E; alt. 818 m; 04–9.VII.2022; Jieling Luo, Chaomei Huang leg.

**Figure 6.**
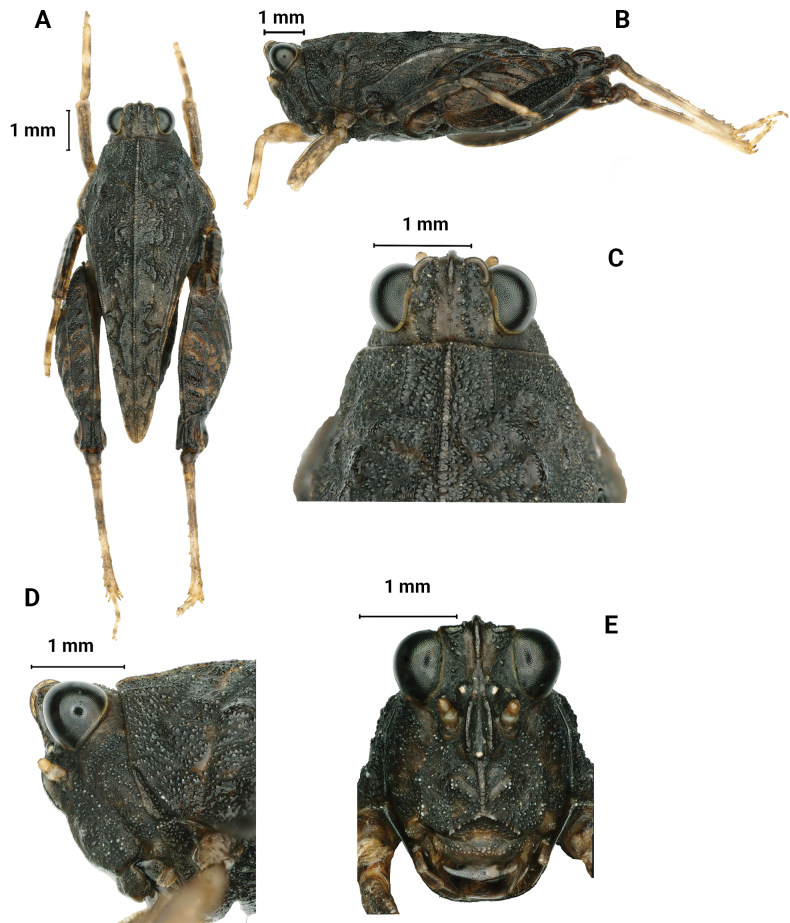
*Macromotettixoidesmaoershanensis* sp. nov., holotype female **A** body, dorsal view **B** the same, lateral view **C** head and anterior part of pronotum, dorsal view **D** the same, lateral view **E** head, frontal view.

**Figure 7.**
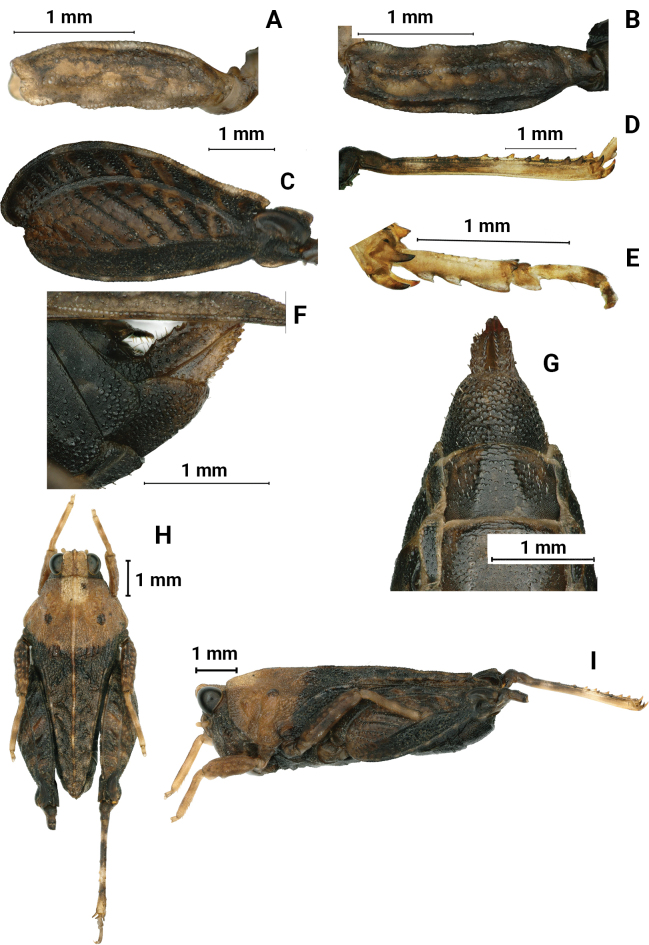
*Macromotettixoidesmaoershanensis* sp. nov., holotype female **A** left fore femur, lateral view **B** left mid femur, lateral view **C** left hind femur, lateral view **D** left hind tibia, lateral view **E** left posterior tarsus, lateral view **F** subgenital plate of female, lateral view **G** subgenital plate of female, ventral view. male, paratype **H** body in dorsal view **I** body in lateral view.

##### Diagnosis.

**Figure 8.**
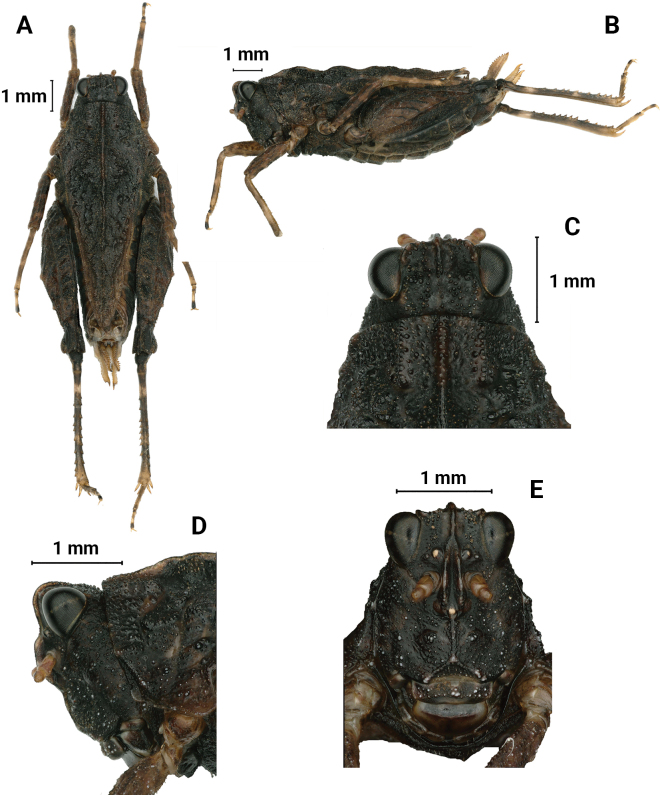
*M.orthomargina*, female **A** body, dorsal view **B** the same, lateral view **C** head and anterior part of pronotum, dorsal view **D** the same, lateral view **E** head, frontal view.

**Figure 9.**
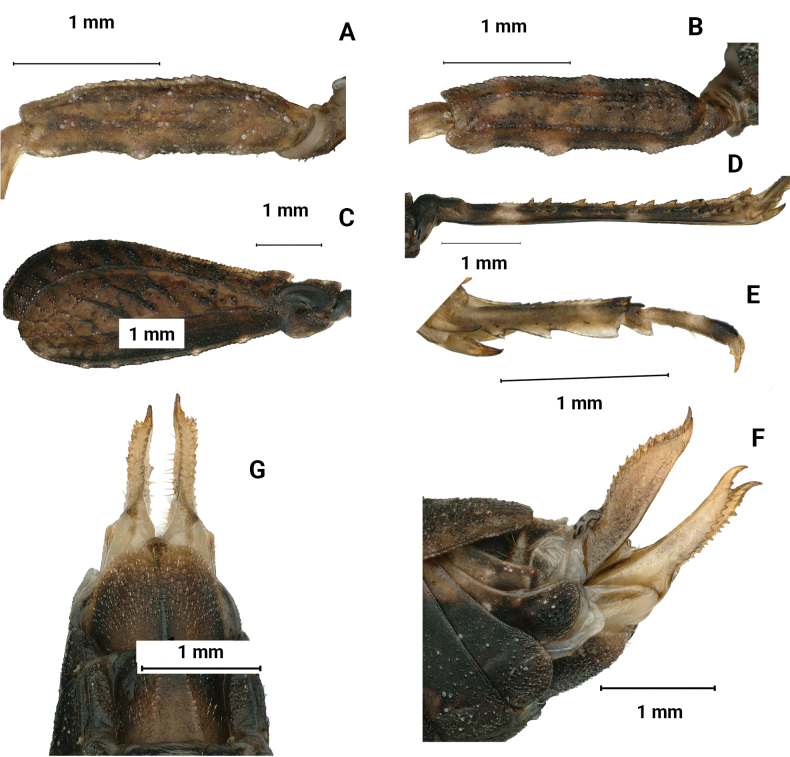
*M.orthomargina*, female **A** left fore femur, lateral view **B** left mid femur, lateral view **C** left hind femur, lateral view **D** left hind tibia, lateral view **E** left posterior tarsus, lateral view **F** subgenital plate of female, lateral view **G** subgenital plate of female, ventral view. male, paratype.

New species is generally similar to *M.orthomargina* (Figs 8, 9) from which it differs in the width of vertex between eyes 2.0× the width of a compound eye (the width of vertex between eyes 3.0× the width of a compound eye in *M.orthomargina*); upper margin of pronotum wide arch-like in lateral view (upper margin of pronotum wavy in lateral view in *M.orthomargina*); the lower margin of hind pronotal process curved (the lower margin of hind pronotal process is straight in *M.orthomargina*); lower outer carina of hind femora smooth and without projection (posteromedian of lower outer carina of hind femora with two or three projections in *M.orthomargina*); lower margin of hind femora serrated (lower margin of hind femora big sawtooth in *M.orthomargina*). It is also similar to *M.undulatifemura*Deng et al. (2012) but differs from the latter by median carina of pronotum slightly arc in profile (median carina of pronotum undulated in profile in *M.undulatifemura*); apex of hind pronotal process rounded (apex of hind pronotal process slightly concave in *M.undulatifemura*); lower margin of hind femora serrated (lower margin of hind femora with five or six teeth and undulated in *M.undulatifemura*).

##### Description.

**Female.** short and small size. Body length 7–8 mm, pronotum length 6–7 mm, hind femur length 4–5 mm.

***Head*.** Head and eyes not exserted above pronotal surface (Fig. 6B). Compound eyes nearly rounded; in dorsal view, width of vertex between eyes 2× width of a compound eye; anterior margin of fastigium nearly straight, surpassing anterior margin of eye; median carina visible anteriorly; vertex uneven with paired fossulae (Fig. 6C). In lateral view, frontal ridge and vertex forming fillet; frontal costa concave between eyes, protruded anteriorly, and wide arc between antennal grooves (Fig. 6D). In frontal view, lateral ocelli are located on both sides of frontal costa, frontal costa bifurcated above lateral ocelli, the bifurcation of the frontal costa in the middle of the compound eye height; longitudinal furrow divergent between antennae, width of longitudinal furrow of frontal ridge 1.3× antennal groove diameter (Fig. 6E). Antennae short, filiform, antennal grooves inserted below inferior margins of compound eyes, 15-segmented, the 10^th^ and 11^th^ segments are the longest, ~ 2.0–3.0× longer than its width. Eyes globose, lateral (paired) ocelli located in lowest third of compound eye height.

***Thorax*.** Pronotum not smooth and has irregular tuberculate (Fig. 6A). In dorsal view, median carina of pronotum obvious, anterior margin of pronotum nearly truncate and not reaching the posterior margin of the compound eye; humeral angle obtuse angle, interhumeral carina visible; hind pronotal process narrow and short, surpassing knee of hind femur and almost reaching apex of hind femur and its apex rounded; In profile (Fig. 6B), median carina of pronotum slightly arch-like; lower margin of hind process curved, external lateral carinae of metazona also slightly curved, width of infrascapular area is 0.8–0.9 mm. Posterior angles of lateral lobes turned downwards, apex of posterior angles obtuse rounded, posterior margins of lateral lobes of pronotum only with ventral sinus and tegminal (upper) sinus absent. Tegmina and hind wings invisible.

***Legs*.** Upper margin of fore and middle femora finely serrated, with carinated, ventral margins undulated (Fig. 7A, B). Hind femora robust and short, 2× as long as wide; with carinated, dorsal margin, and ventral margin finely serrated (Fig. 7C); antegenicular denticles and genicular denticles acute. Outer side and inner side of hind tibia with 5–7 spines (Fig. 7D). First segment of posterior tarsi is 1.5× as long as the third, pulvilli of first segment of posterior tarsi as long as the second, apices of first and second acute, apices of third right angle (Fig. 7E).

***Abdomen*.** Ovipositor narrow and short; upper and lower valvulae with slender saw-like teeth; length of upper valvulae 2.0× its width. Length of subgenital plate 2.5× its width, middle of posterior margin of subgenital plate slightly triangular and projecting (Fig. 7F, G).

***Coloration*.** Body dark brown. Hind tibia yellowish brown, with two light rings in the middle.

**Male.** Similar to female, but smaller and narrower (Fig. 7H, I). Body length 6–8 mm, pronotum length 5–6 mm, hind femur length 4 mm. Width of vertex between eyes 2× width of compound eye. Subgenital plate short conical.

##### Etymology.

The new species was named after the type locality, Maoershan, Guangxi, China.

##### Distribution.

China: Guangxi.

#### ﻿ Macromotettixoides brachycorna sp. nov.

Taxon classificationAnimaliaOrthopteraTetrigidae

96C8B358-CB89-5DAB-994E-3357F2434752

https://zoobank.org/E88A7215-0F52-49E5-B63B-E26F7518BADA

Figs 10 , 11

##### Material examined.

***Holotype***: China ♀; Guangxi Province, Hechi, Huanjiang, Yangmei’ao; 25°11'41"N, 108°38'51"E; alt. 1169.13 m; 03.IX.2021; Chaomei Huang leg. ***Paratypes***: China 1♂1♀; Guangxi, Hechi, Huanjiang, Yangmei’ao; 25°11'41"N, 108°38'51"E; alt. 1169.13 m; 29.VII.2022; Chaomei Huang and Jieling Luo leg.

##### Diagnosis.

The new species is similar to *Macromotettixoidesmaoershanensis* sp. nov. from which it differs in width of vertex between eyes 1.3× width of a compound eye (width of vertex between eyes 2.0× width of a compound eye in *M.maoershanensis*); anterior margin of fastigium not surpassing anterior margin of eye (anterior margin of fastigium surpassing anterior margin of eye in *M.maoershanensis*); median carina of pronotum slightly elevated and undulated in profile (median carina of pronotum slightly arc-like in profile in *M.maoershanensis*); ventral margin of middle femora slightly undulate (ventral margin of middle femora distinctly undulate in *M.maoershanensis*). It is also similar to *Macromotettixoidestuberculata* Mao, Li & Han, 2020 but differs from the latter by width of vertex between eyes 1.3× width of compound eye (width of vertex between eyes 1.7× width of compound eye in *M.tuberculata*); antennal grooves inserted far below inferior margin of compound eyes (antennal grooves inserted between inferior margin of compound eyes); hind pronotal process narrowly rounded (hind pronotal process broad in *M.tuberculata*); lower margin of hind process bend upwards at 1/4 and then tilt straight up, lateral carinae of metazona curved (lower margin of hind process and lateral carinae of metazona slightly straight in *M.tuberculata*).

##### Description.

**Female.** Body size small. Body length 8 mm, pronotum length 6–7 mm, hind femur length 4 mm.

**Figure 10.**
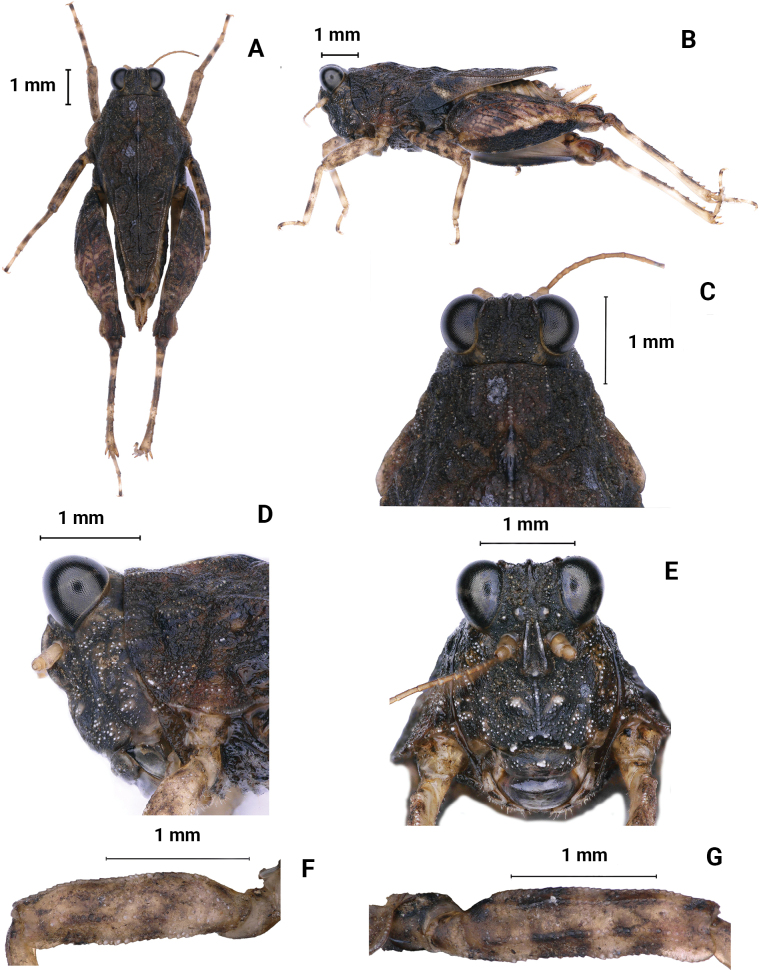
*Macromotettixoidesbrachycorna* sp. nov., holotype female **A** body, dorsal view **B** the same, lateral view **C** head and anterior part of pronotum, dorsal view **D** head, lateral view **E** head and anterior part of pronotum, frontal view **F** left fore femur, lateral view **G** left mid femur, lateral view.

***Head*.** Head and eyes exserted above pronotal surface (Fig. 10B). Face and vertex rough, covered with small granules, not fossulae; medial carina erected in anterior half, but absent in posterior half; vertex 1.3× as wide as a compound eye, not surpassing anterior margin of eyes; anterior margin arc and depressed, curved inward and level with the top of the eyes (Fig. 10C). In lateral view, frontal ridge and vertex forming a rounded-angle shape; eyes oval and not protruding with vertex; frontal costa rounded between antennal grooves (Fig. 10D). In frontal view, frontal costa bifurcated above lateral ocelli, the bifurcation of the frontal costa in the middle of the compound eye height; antennae short, filiform, antennal grooves inserted far below inferior margin of compound eyes, 13-segmented, the 7^th^ and 8^th^ segments are the longest, ~ 3–3.5× longer than its width, antennal grooves 1.5× as wide as diameter of scapus; lateral ocelli placed at lower one third of inner margin of eyes (Fig. 10E).

***Thorax*.** The dorsal surface of the pronotum is coarse dorsum with dense granules, anterior margin of pronotum straight; median carina entire and wavy in profile; lateral carinae of prozona slightly parallel; humeral angle obtuse; hind pronotal process narrow and its apex rounded, reaching pregenicular knee (Fig. 10A); lower margin of hind process bends upwards at 1/4 and then tilt straight up, lateral carinae of metazona curved, width of the area between the two is 0.9 mm (Fig. 10B). Posterior angles of lateral lobes slightly produced outwards, end of posterior angles truncate, posterior margins of lateral lobes of pronotum only with ventral sinus. Tegmina and hind wings invisible.

**Figure 11.**
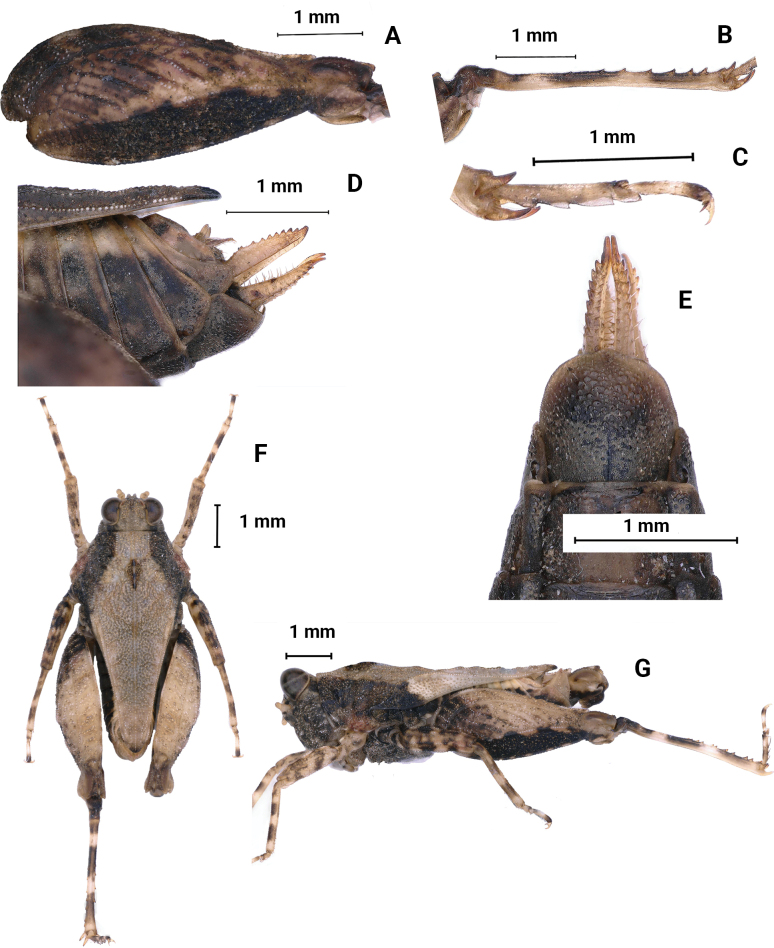
*Macromotettixoidesbrachycorna* sp. nov., holotype female **A** left hind femur, lateral view **B** left hind tibia, lateral view **C** left posterior tarsus, lateral view **D** subgenital plate of female, lateral view **E** subgenital plate of female, ventral view. male, paratype **F** body in dorsal view **G** body in lateral view.

***Legs*.** Fore femora and middle femora with slightly undulated ventral margins (Fig. 10F, G). Hind femora robust and short, 2.3× as long as wide, with carinated and margins finely serrated (Fig. 11A); antegenicular denticles and genicular denticles acute; outer side of hind tibia with 5–7 spines, inner side with five or six spines (Fig. 11B); length of first segment of posterior tarsi slightly longer than third, three pulvilli of first segment of posterior tarsi are increased in turn, three apices acute (Fig. 11C).

***Abdomen*.** Ovipositor narrow and long (Fig. 11D, E), length of upper valvulae 3× its width, upper and lower valvulae with slender saw-like teeth; length of subgenital plate 3.3× its width, middle of posterior margin of subgenital plate triangular projecting.

***Coloration*.** Body dark brown or brown; antennae tawny; hind femur yellowish brown in the middle and dark brown around the sides; hind tibia yellowish brown, with two light rings in the middle.

**Male.** Similar to female, but smaller and narrower (Fig. 11F, G). Body length 6 mm, pronotum length 5 mm, hind femur length 4 mm. Width of vertex between eyes 1.5× width of compound eye. Subgenital plate short, cone-shaped, apex bifurcated.

##### Etymology.

The specific epithet is derived from *brachycorna*, meaning the antennae are shorter and the number of segments is less.

##### Distribution.

China: Guangxi.

## ﻿Discussion

Unfortunately, we do not have access to the species of the South American Tetriginae and Metrodorinae, which makes it impossible to evaluate whether *Systolederus* + *Macromotettixoides* truly belong to the subfamily Metrodorinae. As a result, we cannot provide any conclusive evidence to support their classification within this subfamily. Therefore, further research is needed to clarify their taxonomic status and evolutionary relationships with other Southern American species. But the problematics of the Metrodorinae definition in Asia, especially when differentiated from very diverse Tetriginae, it is often seen that some species of Metrodornae are moved to Tetriginae (Tumbrinck 2019; Subedi 2022). The main reason for this problem is the use of a single character to distinguish between Metrodornae and Tetriginae (the posterior angles of lateral lobes of pronotum produced forward, truncated, or turned downwards, rounded). Typical Metrodorinae are mainly characterized by having the median ocellus and the antenna placed below the eyes, a relatively small divergence of the rami of the frontal costa not forming wide scutellum, and a similar length of the first and third segments of the hind tarsus (Pavón-Gonzalo et al. 2012). Many species of Metrodorinae also share the posterior angles of the lateral lobes of the pronotum produced outwards, often becoming acutely spinose. These characters taken together can separate the subfamily from the other eight subfamilies of Tetrigidae, but single characteristic is not enough to separate itself from the other eight subfamilies (Skejo et al. 2018; Tumbrinck 2019).

*Macromotettixoides* is an apterous genus, but the non-flying Tetrigidae in the Oriental regions have multiple origins. For example, the genus *Hainantettix* Deng, 2020 (Zhang et al. 2020) and the genus *Epitettix* Hancock, 1907 in the subfamily Cladonotinae, as well as the genus *Formosatettix* in the subfamily Tetriginae, are all wingless Tetrigidae that are morphologically similar to *Macromotettixoides*, sometimes making taxonomic identification difficult. At the same time, *Macromotettixoides* is also similar to some brachypronotal and brachypterous Tetriginae such as the genera of *Alulatettix* Liang, 1993 and *Skejotettix* Subedi, 2022. The main differences between them are summarized in Table 7.

**Table 7.** Morphological comparison of *Macromotettixoides*, *Epitettix*, *Hainantettix*, *Formosatettix*, *Alulatettix*, and *Skejotettix*.

Characteristics	*Macromotettixoides*	*Epitettix*	*Hainantettix*	*Formosatettix*	*Alulatettix*	*Skejotettix*
Wing type	apterous	apterous	apterous	apterous	brachypterous	brachypterous
Head	not exserted above the pronotum	not exserted above the pronotum	not exserted above the pronotum	not exserted above the pronotum	not exserted above the pronotum	not exserted above the pronotum
Fastigium of vertex in dorsal view	not surpassing the anterior margin of eyes	distinctly surpassing the anterior margin of eyes	not surpassing the anterior margin of eyes	distinctly surpassing the anterior margin of eyes	not surpassing the anterior margin of eyes	not surpassing the anterior margin of eyes
Fastigium of vertex	vertex not narrowed toward the front	vertex not narrowed toward the front	vertex very strongly narrowed toward the front drawing the eyes together	vertex not narrowed toward the front	vertex not narrowed toward the front	vertex not narrowed toward the front
Width of longitudinal furrow of frontal ridge	narrower than antennal groove diameter	1.3–3.0× antennal groove diameter	1.6–1.8× antennal groove diameter	narrower than antennal groove diameter	narrower than antennal groove diameter	narrower than antennal groove diameter
Tegminal sinus	absent	absent	absent	absent	present	present
Posterior angles of lateral lobes	produced outwards and with truncated apex	produced outwards and with truncated apex	produced outwards and with truncated apex	turned downwards and with rounded apex	turned downwards and with rounded apex	turned downwards and with rounded apex

Since the genus *Macromotettixoides* was erected in 2005, a total of 22 species has been described, another six species transferred to this genus (Zheng et al. 2006, 2009; Zheng and Shi 2009; Deng 2011; Deng et al. 2012, 2014, 2020; Zheng 2013a, 2013b; Zha et al. 2017; Han et al. 2020; Li et al. 2020a; Peng et al. 2021; Fan et al. 2023; Wei and Deng 2023; this study), and two species were moved to *Hainantettix* (Subedi 2022). As a result, 26 species of this genus are now known in the world.

Although the phylogenetic tree in this study supports the genus *Macromotettixoides* being assigned to Tetriginae rather than Metrodorinae, it is limited by the available data and cannot fully confirm its taxonomic placement and monophyletic. Therefore, more comprehensive molecular and morphological data are needed to further investigate the evolutionary relationships and taxonomic status of *Macromotettixoides*.

## Supplementary Material

XML Treatment for Macromotettixoides maoershanensis

XML Treatment for Macromotettixoides brachycorna

## ﻿Additional information

### Author ORCIDs

Jieling Luo https://orcid.org/0000-0002-5657-3436

Rongjiao Zhang https://orcid.org/0000-0001-5545-856X

Weian Deng https://orcid.org/0000-0002-8023-2498

### Data availability

All of the data that support the findings of this study are available in the main text or Supplementary Information.

## Supplementary materials

Supplementary material 1 Mitochondrial genome comparison of 37 species in Tetrigidae, the initiation and termination codons of PCGs of mitogenomes in Tetrigidae Data type docx https://binary.pensoft.net/file/1001330This dataset is made available under the Open Database License (http://opendatacommons.org/licenses/odbl/1.0/). The Open Database License (ODbL) is a license agreement intended to allow users to freely share, modify, and use this Dataset while maintaining this same freedom for others, provided that the original source and author(s) are credited.Jieling Luo, Rongjiao Zhang, Weian Deng
