# Supplementary material for: ﻿First mitogenomic characterization of Macromotettixoides (Orthoptera, Tetrigidae), with the descriptions of two new species
Source: Zookeys. 2024 Mar 14;1195:95–120. doi: 10.3897/zookeys.1195.112623 (PMC10958162; doi:10.3897/zookeys.1195.112623)
Supplement: Supplementary material 1 — Mitochondrial genome comparison of 37 species in Tetrigidae, the initiation and termination codons of PCGs of mitogenomes in Tetrigidae [file zookeys-1195-095_article-112623__-s001.docx]

**Supplementary material**

**Table S1.** Mitochondrial genome comparison of 37 species in Tetrigidae.

| **Species** | **Genome** | | **PCGs** | | **16SrRNA** | | **12SrRNA** | | **CR** | | **tRNAs** | |
| --- | --- | --- | --- | --- | --- | --- | --- | --- | --- | --- | --- | --- |
|  | **Length** | **AT content** | **Length** | **AT content** | **Length** | **AT content** | **Length** | **AT content** | **Length** | **AT content** | **Length** | **AT content** |
| *A. yunnanensis* | 15104 | 75.2 | 11115 | 74.6 | 1340 | 78.4 | 787 | 77.2 | 460 | 80.9 | 1439 | 75.2 |
| *B. lativertex* | 15054 | 73.6 | 9768 | 72.6 | 1295 | 77.3 | 757 | 75.9 | 448 | 77.2 | 1428 | 74.6 |
| *B. sikkinensis* | 14957 | 73.5 | 9801 | 72.7 | 1291 | 77.3 | 753 | 75.7 | 422 | 74.9 | 1429 | 74 |
| *B. yuanbaoshanensis* | 14905 | 73.8 | 10833 | 72.8 | 1297 | 77.5 |  |  |  |  | 1434 | 75.1 |
| *C. longjiangensis* | 14495 | 73.1 | 11100 | 72.1 | 1292 | 77.8 | 563 | 76.2 |  |  | 1410 | 74.7 |
| *C. longtanensis* | 16861 | 74.8 | 11094 | 72.1 | 1302 | 78.5 | 781 | 75.6 | 2083 | 85.8 | 1433 | 74.2 |
| *C. japonicus* | 14247 | 75.6 | 11067 | 75.1 | 1297 | 79.1 | 580 | 73.8 |  |  | 1219 | 76 |
| *E. dorsifera* | 15326 | 72.1 | 11103 | 70.6 | 1293 | 78.5 | 809 | 76.2 | 649 | 76.1 | 1439 | 74.2 |
| *E. serrifemora* | 14947 | 74.7 | 11106 | 73.6 | 1303 | 79.2 | 745 | 77 |  |  | 1384 | 75.3 |
| *E. oculatus* | 14838 | 75.3 | 11085 | 74.1 | 1277 | 79.3 | 563 | 74.8 |  |  | 1218 | 75.4 |
| *E. bimaculatus* | 15221 | 72.8 | 11082 | 71.8 | 1290 | 77 | 790 | 75.8 | 634 | 75.2 | 1436 | 74.3 |
| *E. variabilis* | 15194 | 73.6 | 11082 | 72.5 | 1296 | 77.1 | 801 | 74.7 | 582 | 78.3 | 1444 | 75.3 |
| *F. longicornis* | 15566 | 70.4 | 11112 | 68.8 | 1291 | 75.8 | 778 | 69 |  |  | 1296 | 72.7 |
| *F. qinlingensis* | 15180 | 75.6 | 11094 | 74.7 | 1297 | 79.3 | 781 | 76.7 | 600 | 80.8 | 1430 | 75.3 |
| *L. prominenoculus* | 15025 | 73.5 | 11085 | 72.3 | 1286 | 78.1 | 585 | 71 |  |  | 1213 | 74.7 |
| *M. brachycornis* | 18034 | 71.4 | 11115 | 69.4 | 1285 | 77.1 | 386 | 68.7 |  |  | 1435 | 72.7 |
| *M. maoershanensis* | 16995 | 73.7 | 11091 | 72.3 | 1285 | 76.9 | 695 | 76.9 | 2096 | 79.1 | 1440 | 73.2 |
| *M. orthomarginis* | 16999 | 73.2 | 11100 | 71.2 | 1305 | 77.5 | 794 | 73.6 | 2205 | 80 | 1439 | 72.9 |
| *M. convexa* | 15089 | 74 | 10767 | 72.8 | 1288 | 78.3 | 736 | 74.8 |  |  | 1418 | 74.3 |
| *P. hainanense* | 15307 | 70 | 11187 | 69.2 | 1284 | 73.4 | 764 | 72 | 577 | 68.5 | 1429 | 72.6 |
| *P. sichuanense* | 17849 | 74.2 | 11118 | 71.5 | 1295 | 76.1 | 739 | 72.8 | 3290 | 83.2 | 1426 | 72.8 |
| *S. borneensis* | 13112 | 71.7 | 11106 | 71.2 | 847 | 73.6 |  |  |  |  | 1160 | 74.5 |
| *S. melli* | 16006 | 70.8 | 11100 | 69.2 | 1312 | 75.6 | 742 | 72.7 |  |  | 1437 | 70.7 |
| *S. anhuiensis* | 14598 | 68.2 | 11124 | 67 | 1296 | 73.8 | 765 | 71.3 |  |  | 1431 | 71.2 |
| *S. bashanensis* | 15262 | 75.7 | 11022 | 75.2 | 1293 | 79.5 | 803 | 76.8 | 695 | 78.4 | 1436 | 74.8 |
| *S. hainanensis* | 15458 | 74 | 11070 | 73.1 | 1307 | 79 | 740 | 76.4 |  |  | 1430 | 73.5 |
| *S. nigropennis* | 14775 | 74 | 11034 | 73 | 1305 | 78.8 | 779 | 76.9 |  |  | 1440 | 73.7 |
| *S. spicupennis* | 14946 | 76.2 | 11088 | 75.6 | 1293 | 79.5 | 781 | 77.5 |  |  | 1457 | 75.1 |
| *T. japonica* | 14652 | 73.8 | 10068 | 72.4 | 1313 | 77.2 | 744 | 76.3 |  |  | 1431 | 73.6 |
| *T. ruyuanensis* | 15128 | 75.6 | 11106 | 74.8 | 1290 | 79 | 786 | 76.9 | 531 | 82.6 | 1440 | 75 |
| *T. nodulosa* | 15218 | 75.5 | 11091 | 74.7 | 1298 | 78.9 | 785 | 76.8 | 619 | 79.6 | 1434 | 75.4 |
| *T. obtusilobata* | 14759 | 71.8 | 11091 | 70.5 | 1283 | 76.9 | 665 | 72.5 |  |  | 1284 | 74.1 |
| *T. yunnana* | 14538 | 71.8 | 11091 | 70.6 | 1292 | 76.8 | 558 | 72.6 |  |  | 1242 | 74.5 |
| *T. bufo* | 17859 | 73.6 | 11097 | 70.6 | 1300 | 76.1 | 744 | 73 | 2947 | 82.8 | 1421 | 74 |
| *T. tonkinensis* | 14578 | 71.6 | 11091 | 70.6 | 1340 | 77 | 729 | 74.5 |  |  | 1382 | 72.5 |
| *Z. curvispinus* | 16696 | 68.6 | 11139 | 66.8 | 1205 | 71.9 | 737 | 70 |  |  | 1427 | 69.2 |
| *A. yunnanensis* | 16380 | 76.1 | 11112 | 74.8 | 1134 | 76.7 | 781 | 76.8 |  |  | 1221 | 76.6 |

**Table S2.** The initiation codons of PCGs of mitogenomes in Tetrigidae.,

| **Species** | **Initiation codon** | | | | | | | | | | | | |
| --- | --- | --- | --- | --- | --- | --- | --- | --- | --- | --- | --- | --- | --- |
|  | ***atp6*** | ***atp8*** | ***cox1*** | ***cox2*** | ***cox3*** | ***cytb*** | ***nad1*** | ***nad2*** | ***nad3*** | ***nad4*** | ***nad4L*** | ***nad5*** | ***nad6*** |
| *A. yunnanensis* | ATG | ATG | ATC | ATG | ATA | ATG | ATA | ATG | ATA | ATG | ATT | ATG | ATG |
| *B. lativertex* | ATG | ATG | ATC | ATG | ATG | ATG | ATT | ATT | ATT | N/A | ATT | ATG | TTG |
| *B. sikkinensis* | ATG | ATG | ATC | ATG | ATG | ATG | ATT | ATT | ATT | N/A | ATT | ATG | TTG |
| *B. yuanbaoshanensis* | ATG | ATG | ATC | ATG | ATG | ATG | ATT | ATT | ATT | ATC | N/A | ATG | TTG |
| *C. longjiangensis* | ATG | ATG | ATC | ATG | ATG | ATG | ATT | GTG | ATA | ATG | ATT | ATG | ATG |
| *C. longtanensis* | ATG | ATG | ACA | ATG | ATA | ATG | ATT | GTG | ATA | ATG | TTG | ATG | ATG |
| *C. japonicus* | ATG | ATG | AAA | ATG | ATG | ATG | ATT | ATT | ATT | ATG | ATT | ATG | TTG |
| *E. dorsifera* | ATG | ATG | ATC | ATG | ATA | ATG | ATT | ATG | ATC | ATG | ATT | ATG | ATT |
| *E. serrifemora* | ATG | ATG | ATC | ATG | ATG | ATG | ATT | ATT | ATC | ATG | ATT | ATG | TTG |
| *E. oculatus* | ATG | ATA | AAA | ATG | ATG | ATG | ATT | ATC | ATT | ATG | ATT | ATG | TTG |
| *E. bimaculatus* | ATG | ATG | ATC | ATG | ATA | ATG | ATT | GTG | ATC | ATG | ATT | ATG | ATT |
| *E. variabilis* | ATG | ATG | ATC | ATG | ATA | ATG | ATT | GTG | ATT | ATG | ATT | ATG | ATG |
| *F. longicornis* | ATG | ATG | AAA | ATG | ATG | ATG | ATT | ATA | ATA | ATG | ATT | ATG | ATG |
| *F. qinlingensis* | ATG | ATG | ATC | ATG | ATG | ATG | ATT | ATG | ATT | ATG | ATT | ATG | ATG |
| *L. prominenoculus* | ATG | ATG | AAA | ATG | ATG | ATG | ATT | ATC | ATC | ATG | ATT | ATG | TTG |
| *M. brachycornis* | ATG | ATG | ATC | ATG | ATG | ATG | ATT | ATA | ATC | ATG | ATT | ATG | ATG |
| *M. maoershanensis* | ATG | ATG | ATC | ATG | ATG | ATG | ATT | ATT | ATA | ATG | ATT | ATG | ATA |
| *M. orthomarginis* | ATG | ATG | ATC | ATG | ATG | ATG | ATA | ATT | ATA | ATG | ATT | ATG | ATA |
| *M. convexa* | ATG | ATA | ATC | ATG | ATG | ATG | ATT | ATC | ATT | ATG | N/A | ATG | TTG |
| *P. hainanense* | ATG | ATG | ATC | ATG | ATG | ATG | ATT | ATT | ATT | ATG | ATT | TTG | GTG |
| *P. sichuanense* | ATG | ATG | AAA | ATG | ATG | ATG | ATT | ATT | ATT | ATG | ATT | TTG | ATG |
| *S. borneensis* | ATG | ATG | ATC | ATG | ATG | ATG | ATT | ATG | ATC | ATG | ATT | ATT | ATT |
| *S. melli* | ATG | ATG | ATC | ATG | ATG | ATG | ATT | ATC | ATT | ATG | ATT | ATG | TTG |
| *S. anhuiensis* | ATG | ATG | ATC | ATG | ATA | ATG | ATT | ATT | ATA | ATA | ATT | ATG | TTG |
| *S. bashanensis* | ATG | ATG | ATC | ATG | ATA | ATG | ATT | ATA | TTG | ATG | ATT | ATT | TTG |
| *S. hainanensis* | ATG | ATG | ATC | ATG | ATG | ATG | ATT | ATG | ATT | ATG | ATT | ATG | ATT |
| *S. nigropennis* | ATG | ATG | ATC | ATG | ATG | ATG | ATT | ATG | ATT | ATG | ATT | ATG | ATT |
| *S. spicupennis* | ATG | ATG | ATC | ATG | ATA | ATG | ATT | N/A | TTG | ATG | ATT | ATG | TTG |
| *T. japonica* | ATG | ATG | ATC | ATG | ATA | ATG | ATA | ATG | ATA | ATG | ATT | ATG | ATG |
| *T. ruyuanensis* | ATG | ATG | ATC | ATG | ATA | ATG | ATT | ATG | ATC | ATG | ATT | ATG | ATG |
| *T. nodulosa* | ATG | ATG | AAA | ATG | ATG | ATG | ATT | ATT | ATC | ATG | ATT | ATG | TTG |
| *T. obtusilobata* | ATG | ATG | ATC | ATG | ATG | ATG | ATT | ATT | ATA | ATG | ATT | ATG | TTG |
| *T. yunnana* | ATG | ATG | ATC | ATG | ATG | ATG | ATT | ATT | ATC | ATG | ATT | ATG | TTG |
| *T. bufo* | ATA | ATG | ACA | ATA | ATG | ATG | ATT | ATG | ATT | ATT | ATT | ATG | TTG |
| *T. tonkinensis* | ATG | ATA | ATC | ATA | ATG | ATG | ATT | ATC | ATT | ATG | TTG | TTG | ATT |
| *Z. curvispinus* | ATG | ATG | AAA | ATG | ATG | ATG | ATT | ATT | ATT | ATG | ATT | ATG | ATC |

**Table S3**. The termination codons of PCGs of mitogenomes in Tetrigidae.

| **Species** | | **Terminal codon** | | | | | | | | | | | | |
| --- | --- | --- | --- | --- | --- | --- | --- | --- | --- | --- | --- | --- | --- | --- |
|  |  | ***atp6*** | ***atp8*** | ***cox1*** | ***cox2*** | ***cox3*** | ***cytb*** | ***nad1*** | ***nad2*** | ***nad3*** | ***nad4*** | ***nad4L*** | ***nad5*** | ***nad6*** |
| *A. yunnanensis* | TAA | TAA | TAA | T | TAA | T | TAA | T | TAG | TA | TAA | T | TAA |  |
| *B. lativertex* | TAA | TAA | T | TAA | TAG | TAA | TAA | TAA | TAG | N/A | TAA | T | TAA |  |
| *B. sikkinensis* | TAA | TAA | T | TAA | TAG | TAA | TAA | TAA | TAG | N/A | TAA | T | TAA |  |
| *B. yuanbaoshanensis* | TAA | TAA | T | TAA | TAG | TAA | TAA | TAA | TAG | TA | N/A | T | TAA |  |
| *C. longjiangensis* | TAA | TAA | T | TAA | T | TAA | TAA | TAA | T | TAG | TAA | T | TAA |  |
| *C. longtanensis* | TAA | TAA | T | TAA | TAA | TAA | TAA | TAA | TAG | TAG | TAA | T | TAA |  |
| *C. japonicus* | TAA | TAA | T | TAA | TAA | TAA | TAA | TAA | TAA | TAG | TAA | TA | TAA |  |
| *E. dorsifera* | TAA | TAA | T | TAA | T | TAG | TAG | TAA | TAG | TAG | TAA | T | TAA |  |
| *E. serrifemora* | TAA | TAA | T | TAA | T | TAG | TAA | TAA | TAG | TAG | TAA | T | TAA |  |
| *E. oculatus* | TAA | TAA | T | TAA | TAG | TAA | TAA | TAA | TAG | TAA | TAA | T | TAA |  |
| *E. bimaculatus* | TAA | TAA | T | TAA | T | TAG | TAA | TAA | TAG | TAG | TAA | T | TAA |  |
| *E. variabilis* | TAA | TAA | T | TAA | T | TAG | TAA | TAA | TAG | TAG | TAA | T | TAA |  |
| *F. longicornis* | TAA | TAA | T | TAA | TAA | TAG | TAA | TAA | TAG | TAG | TAA | T | TAA |  |
| *F. qinlingensis* | TAA | TAA | T | TAA | T | TAG | TAA | TAA | T | TAG | TAA | T | TAA |  |
| *L. prominenoculus* | TAA | TAA | T | TAA | TAA | TAA | TAA | TAA | TAG | TA | TAA | TAA | TAA |  |
| *M. brachycornis* | TAA | TAA | TAA | TAA | T | TAA | TAA | TAA | TAG | TAA | TAA | TA | TAA |  |
| *M. maoershanensis* | TAA | TAA | TAA | TAA | T | TAA | TAA | TAA | TAG | TAG | TAA | T | TAA |  |
| *M. orthomarginis* | TAA | TAA | TAA | TAA | T | TAA | TAA | TAA | TAG | TAG | TAA | T | TAA |  |
| *M. convexa* | TAA | TAA | T | TAA | TAA | TAA | TAA | TAA | TAG | TAG | N/A | T | TAA |  |
| *P. hainanense* | TAA | TAA | T | TAA | T | TAG | TAA | TAA | TAG | TAG | TAA | T | TAA |  |
| *P. sichuanense* | TAA | TAA | T | TAA | T | TAG | TAA | TAA | TAG | TAG | TAA | T | TAA |  |
| *S. borneensis* | TAA | TAA | T | TAA | T | TAG | TAG | TAA | TAG | TAG | TAA | T | TAA |  |
| *S. melli* | TAA | TAA | T | TAA | TAA | TAG | TAA | TAA | TAG | TAA | TAA | T | TAA |  |
| *S. anhuiensis* | TAA | TAA | TAA | TAA | TAA | TAA | TAA | TAA | TAG | TAG | TAA | T | TAA |  |
| *S. bashanensis* | TAA | TAA | T | TAA | T | TAA | TAA | TAA | TAG | TAG | TAA | T | TAA |  |
| *S. hainanensis* | TAA | TAA | T | TAA | T | TAA | TAA | TAA | TAG | TAG | TAA | T | TAA |  |
| *S. nigropennis* | TAA | TAA | T | TAA | T | T | TAA | TAA | TAG | TAG | TAA | T | TAA |  |
| *S. spicupennis* | TAA | TAA | T | TAA | T | TAA | TAA | N/A | TAG | TAG | TAA | T | TAA |  |
| *T. japonica* | TAA | TAA | TAA | TAA | T | TAG | TAA | TAA | TAG | TAG | TAA | TAA | TAA |  |
| *T. ruyuanensis* | TAA | TAA | T | TAA | T | TAG | TAA | TAA | TAG | TAG | TAA | T | TAA |  |
| *T. nodulosa* | TAA | TAA | T | TAA | TAA | TAA | TAA | TAA | TAG | TAG | TAA | T | TAA |  |
| *T. obtusilobata* | TAA | TAA | T | TAA | T | TAA | TAA | TAA | T | TAG | TAA | T | TAA |  |
| *T. yunnana* | TAA | TAA | T | TAA | TAA | TAA | TAA | TAA | TAG | TAG | TAA | T | TAA |  |
| *T. bufo* | TAA | TAA | T | TAA | T | TAG | TAG | TAA | TAG | TAG | TAA | T | TAA |  |
| *T. tonkinensis* | TAA | TAA | T | TAG | TAA | TAG | TAA | TAA | TAG | TAG | TAG | T | TAA |  |
| *Z. curvispinus* | TAA | TAA | T | TAA | T | TAA | TAA | TAA | TAG | TAA | TAA | T | TAA |  |
